# Utility of Matrix-Assisted Laser Desorption Ionization Time-of-Flight Mass Spectrometry in Periprosthetic Joint Infection Diagnosis

**DOI:** 10.7759/cureus.70650

**Published:** 2024-10-01

**Authors:** Emanuel-Cristian Sandu, Adrian Cursaru, Sergiu Iordache, Bogdan Serban, Mihai Aurel Costache, Catalin Cirstoiu

**Affiliations:** 1 Orthopedics and Traumatology, Faculty of Medicine, University of Medicine and Pharmacy "Carol Davila", Bucharest, ROU; 2 Orthopedics and Traumatology, University Emergency Hospital, Bucharest, ROU

**Keywords:** differential diagnoses, direct maldi-tof-ms, maldi-tof-ms, orthopedic implant related infection, prosthetic joint infection (pji)

## Abstract

One of the most feared complications of arthroplasty surgery is septic loosening. Periprosthetic joint infection (PJI) requires an accurate and fast diagnosis, and identification of pathogen microorganisms is essential for successful treatment. While standard bacteriological cultures can identify bacteria in seven to 14 days with sensitivity ranging from 35% to 70% that could further be increased by sonication of the explanted prosthesis, we would like to review a more novel and faster method of PJI detection and bacterial identification. Matrix-assisted laser desorption ionization time-of-flight mass spectrometry (MALDI-TOF/MS) is a technique that identifies bacteria based on peptides and protein ions from the cell surface, comparing the obtained results within a database. While MALDI-TOF/MS is not a novel method, being already successfully used in microbiology, its role in PJI is still being researched. With this paper, we would like to reveal the current state of development in implementing MALDI-TOF/MS as an alternative or auxiliary test to classic bacterial cultures in orthopedic implant infectious pathology to increase the accuracy of detecting and identifying bacteria.

## Introduction and background

With the great success of knee and hip arthroplasties, the total number performed worldwide is increasing fast, resulting in an also significant increase in revision surgeries [1]. The most common arthroplasty failure reasons can be aseptic loosening, instability, periprosthetic fracture, or infection of the prosthesis. Even though the incidence of periprosthetic joint infection (PJI) is lower than aseptic loosening, the condition is a far more serious and complex complication. The incidence of PJI on primary arthroplasty is around 0.2%-1.1% of revision surgeries but can reach 5% after revision of the primary prosthesis [2]. Management and diagnosis of PJI are still challenging today for the healthcare system. Orthopedic surgeries that require implanting different types of materials to cure disabling disorders or even fractures, with the addition of a foreign body, will always be susceptible to bacterial colonization. The surface of the implants is a perfect site for colonization and bacteria will develop a biofilm that will drastically affect the accuracy of diagnosis and the penetrability of antibiotics. In a need to get a reliable and good result for PJI detection before revision surgery, a customized combination of diagnostic tests has been promoted. Almost all infection societies, like the European Bone and Joint Infection Society (EBJIS), the Infectious Diseases Society of America (IDSA), and the Musculoskeletal Infection Society (MSIS) reached an agreement that the presence of a sinus tract communicating with the prosthesis or the presence of two positive microbiological cultures identifying the same pathogen are major criteria for PJI. Even though the specificity of those criteria is 100%, the sensitivity is still low, and so, in addition to those, more tests were proposed as diagnostic tools like conventional radiographs, erythrocyte sedimentation rate (ESR), C-reactive protein (CRP), synovial fluid aspiration for counting and identifying cells and further microbiology testing, histology examination of the periprosthetic membrane.

Prompt and fast identification of the etiological pathogen is the key and indispensable requirement for a successful treatment plan since different therapeutic routes can only be proposed if the bacterial species responsible for the infectious process is identified, as well as its antibiotic resistance, virulence potential, and its ability to form biofilm on the surface of the implant.

The most commonly used tests to diagnose PJI start with the classical in vitro culture inoculated with collected samples from periprosthetic tissues or synovial fluid to identify the pathogen bacteria.

We have to mention an important aspect of this type of culture. The method was initially developed for acute infections caused by planktonic bacteria, so, in a pathology that gravitates around biofilm-related infections of the implants where bacteria adhere to the implant and evolve in a more latent state, we can see the reason why the reliability of this kind of test is uncertain. A review by Costerton et al. [3] shows that pathogen identification for biofilm-related infections is difficult mostly because of the frequent failures in detaching and collecting biofilm cells from tissue samples and further inoculating them on agar plates. Unlike free bacteria that produce colonies on agar, bacteria from biofilm do not. Failing to identify a pathogen of PJI often leads to misdiagnosis, resulting in an inappropriate therapeutic option with major consequences on the prognosis and evolution of the patients, septic and aseptic revision involving different treatment pathways.

The new emergent technologies for rapid bacteriological detection and identification show a shift from the classic biochemical and molecular testing toward those using mass spectrometry (MS) for the semiquantitative analysis of bacterial proteins and genetic structure. The most recent technologies are PCR-electrospray ionization (ESI)/MS - Ibis [3] and matrix-assisted laser desorption ionization time-of-flight mass spectrometry (MALDI-TOF/MS). Diagnostic sensitivity and specificity, as well as reproducibility and costs, are discussed in research conducted by Kaleta et al. [4]. The most appealing aspect of those technologies is the rapid identification of the causative bacteria. By removing a subculture step and directly identifying from clinically gathered samples, the time for detection can be reduced once again. Direct identification is possible thanks to novel molecular biology methods such as polymerase chain reaction (PCR) or next-generation sequencing (NGS) [5]. These technologies, while getting excellent results in PJI diagnostics, are still expensive, require bioinformatics expertise, and are not affordable in every clinical laboratory, whereas MALDI-TOF/MS is available in most clinical microbiology departments [6].

This review aims to highlight the utility of the MALDI-TOF/MS technique in the infectious pathology of orthopedics implants. We would like to further present the positive results and the limitations of this method when used as an ancillary test in PJI diagnosis from the few studies that have been published.

## Review

Methodology

PubMed database was searched using the keywords “MALDI-TOF AND (PJI OR Prosthetic Joint Infection).” The search revealed 34 studies in the timeframe 2010-2024 that were assessed by manually reading them and further filtering was done. Inclusion criteria were prospective or retrospective cohort studies that used MALDI-TOF/MS as an auxiliary or alternative test for prosthetic infection detection or bacterial identification in patients who underwent arthroplasty revision surgery. Exclusion criteria were represented by studies where the term "MALDI-TOF/MS" was mentioned in the text but actual testing of the patients using this method was not conducted, case reports and case series studies, and review articles. The final selection included only five studies that assessed the utility of MALDI-TOF/MS as a diagnostic and bacterial identification method in periprosthetic joint infections.

Current diagnostic methods

Clinical Signs

In the case of acute infection (less than one month after prosthesis implantation) there is a higher probability of local clinical signs like pain, edema, erythema, wound dehiscence, or impaired joint function to be present. Some cases may present fever as well. Chronic infections can evolve without any of those clinical signs, with most of the patients presenting only chronic pain, symptoms that can be present in the case of aseptic loosening as well. The only pathognomonic signs for PJI are the presence of a sinus tract or purulence around the prosthesis [7].

Radiological Diagnosis

The sensitivity of plain radiographs is low for PJI detection. Radiological changes (osteolysis or osteopenia - bone areas with lower intensity, periosteal reaction) can be identified in chronic infections. While focal osteolysis around the prosthesis can be present in both septic and aseptic loosening, the presence of this sign in the first three years after surgery could indicate a higher chance of PJI [8]. Another useful imaging technique in evaluating bone loss and the condition of adjacent soft tissue is computed tomography [9]. A good resolution can be obtained using special scanning protocols to reduce implant artifacts. Soft tissue visualization can be further improved by magnetic resonance imaging (MRI).

Bone scintigraphy with 99mTc shows promising sensitivity in detecting PJI, but specificity is still low [10]. With the continuous bone metabolism surrounding the prosthesis in the first two, or three years after implantation, the positive uptake of radioisotope is normal, differential diagnosis at this stage between septic and aseptic loosening cannot be made. Further accuracy improvement can be made by using another radioisotope like Indium-111-labeled leukocytes [11]. Positron emission tomography (PET) using 18-fluorodeoxyglucose could be a more expensive alternative in detecting PJIs with a reported specificity of 86.6% and sensitivity of 82.1% [12].

Laboratory Findings

While serum biomarkers like C-reactive protein (CRP), white blood cell count (WBC), erythrocyte sedimentation rate (ESR), or procalcitonin (PCT) remain one of the most used screening tools for PJIs, their sensitivity and specificity are not high enough to diagnose or rule out PJI by their own. Elevation of serum CRP over 10 mg/L and 30 mm/H for ESR could indicate a possible infection [13]. Clinicians should take into consideration that elevated values of those biomarkers could be present in other inflammatory diseases like autoimmune diseases, malignancies, or simultaneous infections. Commonly, chronic low-grade infections could present normal values of those biomarkers so caution should be advised when interpreting them [14].

Synovial Fluid Biomarkers

One of the most useful tools in PJI diagnosis remains the preoperative joint aspiration of synovial fluid with further fluid analysis. Leukocyte count and granulocyte percentage of synovial fluid is a simple, fast, and accurate test for PJI identification. Values above 2,000 leukocytes/μL and 70% granulocytes are strong indicators of PJI [15].

The increase of synovial CRP has been proven to be more accurate than serum CRP [16]. In a publication where data from 66 patients undergoing revision surgery of the knee arthroplasty was analyzed, the sensitivity of synovial CRP was 84% compared with 76% of the serum CRP, and the specificity was 97% compared with 93% [17].

Leukocyte esterase, an enzyme secreted by neutrophils that are present in the synovial fluid of septic joints has been proposed as a biomarker in detecting PJI [18]. A fast calorimetric strip has been successfully used in other fluids like urine or pleural fluid to detect infection. The accuracy of leukocyte esterase was assessed by a study involving 108 patients who underwent revision surgery of the knee arthroplasty, yielding a sensitivity of 80.6% and specificity of 100% [19].

Another biomarker present in the synovial fluid in case of infection is human α-Defensin, an antimicrobial peptide secreted by the local neutrophils as a part of the innate immune response. Detection of this antimicrobial peptide is already available via laboratory-based enzyme-linked immunosorbent assay (ELISA) or the fast lateral flow test kit. Recent meta-analyses show an accuracy increase for the ELISA test compared with the lateral flow test [20]. The sensitivity of α-defensin detection using the ELISA method in diagnosing PJI can be as high as 97% with a specificity of 97% [21].

Tissue Biopsy Culture

In the cases where PJI is suspected and cultures inoculated with synovial fluid did not manage to identify the causative bacteria, further tests must be performed. While intra-operative culture remains one of the most frequently used methods of bacterial identification considering the vast availability of the technology, the sensitivity ranges between 65% to 94% [22]. As a general rule, at least three to five samples should be collected for culture, and the incubation period should be extended to 14 days for anaerobic cultures [23]. To reduce the false negative results of this method in detecting PJIs, any antibiotic therapy should be discontinued for two weeks before biopsy [24].

Sonication of the Removed Implant

Considering that periprosthetic joint infection is a biofilm-related infection, we can understand the low sensitivity of standard bacterial identification methods. Using ultrasound waves to detach biofilm micro-organisms from the surface of the implants is a method that can further facilitate the identification of causative bacteria. Inoculation of the resulting sonication fluid can be performed onto aerobic and anaerobic plates or blood culture bottles which may further increase the accuracy of the test [25]. Compared to standard cultures of periprosthetic tissue, inoculation of the sonication fluid showed a higher sensitivity (54% compared to 79%) in diagnosing PJI [26].

Histopathological Examination of Periprosthetic Tissue

Considered by many authors as one of the most important criteria for PJI diagnosis, histopathological examination shows a high sensitivity of 95% and specificity of 92%. Analyzed periprosthetic tissue was classified into four types, wear particle disease, infectious type, combined type (first and second types), and indeterminate type [27]. The cut-off point for the number of inflammatory cells visible per high power field of magnification of 400 can vary from more than 1 to more than 10 neutrophils to diagnose PJI [7]. Further immunohistochemistry study can be performed using CD15 marker to improve the visibility of neutrophils. While histopathological examination of the periprosthetic tissue has high accuracy in detecting PJI, it has no role in bacterial identification.

Molecular Diagnosis

Polymerase chain reaction (PCR) can be used to search for 16s ribosomal RNA gene in the synovial fluid or sonication fluid to directly detect most bacteria without a culture. Sensitivity and specificity can be as high as 81% and 96% when using sonication fluid and 84% and 89% when using synovial fluid [28].

Another emerging technology used for PJI diagnosis with great accuracy is metagenomic next-generation sequencing. It can detect the entire bacterial DNA or RNA sequence from a sample and further identify the pathogen based on it [29]. While the accuracy of these tests is high, the limitations of these sensitive methods are still the high costs and susceptibility to false-positive results [30].

MALDI-TOF/MS

MALDI-TOF/MS is a technique that uses soft laser ionization on intact bacteria or bacterial extracts to detect peptide and protein ions of the bacteria cell surfaces according to their relative charges and masses, resulting in bacterial identification, knowing that every bacteria species has a different spectrum based on their mass/charge ratio [31]. Various algorithms to detect species-specific conserved peaks, which are then used as biomarkers to identify the bacterial species have been elaborated. The multiple spectra obtained using those algorithms led to database creation. The most known and used database is the one founded by Bruker Daltonics GmbH, Bremen, Germany, the Bruker MALDI biotype database, which has over 3000 well-defined microbial species [32].

This technique is an important tool in identifying with great success all pathogens that are tested in clinical microbiology laboratories, with some exceptions like viruses and parasites [33]. However, MALDI-TOF/MS was developed and approved for use with pure, isolated colonies, not for identification and detection of pathogens contained directly within primary specimens. While trying to use MALDI-TOF/MS directly from the samples, the clinical laboratory may hinder correct identification and lose all the advantages of the technology. Various outcomes can be encountered like the less probability of identification of some or all bacteria in the case of a multiple microorganism infection [34]. In the case of multiple identification, the confidence level is lower. It can identify the most abundant bacteria only or no identification at all. Another issue that can arise while trying to use direct identification is the overabundance of proteins from human cellular structures in the sample. Even though the current commercial MALDI-TOF/MS tools analyze spectra from proteins ranging from 2000 Da to 20000 Da, they do not discriminate between which proteins from the sample are analyzed. As a result, the sensitivity can drop. To counter this problem, clinical laboratories developed differential centrifugation methods to remove as much as possible of the human cellular material [35].

MALDI-TOF/MS also shows good potential in differentiating within a species, since it can provide a subtyping by improving the algorithm used for comparing different spectra [36]. A study published by Dhiman et al. analyzed the costs and accuracy of different conventional and molecular identification methods and showed that MALDI-TOF/MS is the most cost/efficient technique (0.50$ per specimen for MALDI-TOF/MS compared with 6.86$ for API-Staph or 20.02$ for NGS) and time convenient (0.64 hours total turnaround time per specimen with MALDI-TOF/MS vs. 48-72 h for API-Staph vs. 3.5 h for NGS) [37].

Direct bacterial identification from blood culture bottles using MALDI-TOF/MS in PJI

Even though there are only a few studies that analyze the utility of MALDI-TOF/MS in PJI diagnosis, a prospective multicentric cohort study published in 2023 by Beguiristain et al. [38] assessed the use of MALDI-TOF/MS in improving the sensitivity and specificity of more commonly used diagnostic techniques, and most importantly the time required to achieve a positive result. It included 107 arthroplasty revision cases, from whom 36 cases were confirmed as PJIs using EBJIS definition criteria. They analyzed data from four types of diagnostic methods concluding the following sensitivities and specificities using a confidence interval of 95%, 53% sensitivity and 100% specificity for classic periprosthetic tissue samples culture, 64% sensitivity and 100% specificity for sonication fluid culture obtained after sonication of the explanted prosthesis, 78% sensitivity and 94% specificity for blood culture bottles inoculated with sonication fluid (BCB-SF), and 69% sensitivity and 94% specificity for MALDI-TOF mass spectrometry from positive BCB-SF [38]. We can observe that the highest value for sensitivity was obtained using the BCB-SF method, 78% compared to only 53% for the classic periprosthetic tissue sample cultures. For second place we have the subject of the study, MALDI-TOF/MS from the sonication fluid inoculated BCB with a sensitivity of 69%, still higher than the periprosthetic tissue cultures. Unfortunately, the specificity of this method is slightly lower than the conventional cultures (94% vs. 100%).

In today's treatment protocol dynamics, the need for a fast and safe diagnostic is key for a good patient outcome. In our opinion, the most appealing factor of the study that favors the described technique is the speed at which it can provide positive results. The inoculation of BCB-SF detected an overall 81% of infections and identified 88% of them in the first 24 hours of incubation. Furthermore, applying direct MALDI-TOF/MS from the positive BCB-SF cultures, the pathogen can be identified within the first 48 hours from the surgery except if the pathogen is Cutibacterium acnes, MALDI-TOF/MS not being able to identify it. Being able to get good, reliant results of MALDI-TOF/MS from positive BCB-SF still involved the use of conventional tissue and sonication fluid cultures because of the inability of the technique to identify pathogens in the case of a polymicrobial infection or C. acnes [38].

The study concluded that MALDI-TOF/MS from positive sonication fluid inoculated BCB is a straightforward, fast, and cheap diagnostic method that can drastically improve the accuracy in the diagnosis of PJIs in laboratories where more complex and expensive molecular biology techniques are not available.

Another similar study published by Kuo et al. [39] in 2020 wanted to compare the results of direct MALDI-TOF/MS in contrast with routine MALDI-TOF/MS and conventional cultures in the identification of involved micro-organisms in PJI. Another aim of the study was to assess the time needed for the proposed methods for preliminary strain identification. The prospective study included 77 patients (80 joints) who were diagnosed with PJI using the International Consensus Meeting criteria. Compared with the previews described study that used sonication fluid of the explanted prosthesis to inoculate blood culture bottles, the current one used synovial joint fluid that was aspirated before arthrotomy in the operation room. The collected aspirates were inoculated in 3 types of cultures, aerobic and anaerobic BCB, conventional swabs, and cytology analysis (WBC count and polymorphonuclear leukocyte percentage).

From the 80 cases of PJI, routine and direct MALDI-TOF/MS from the BCB identified 64 positive cultures (80%) compared to only 47 identified by the conventional culture (59%). Further analysis of the types of identified organisms was needed to see which factors influenced the false negative results. Direct MALDI-TOF/MS successfully identified 85.3% of the gram-positive bacteria, in most part Staphylococcus aureus and epidermidis, and 92.3% of gram-negative organisms. No fungal infections were identified by this method. Seventeen samples were marked as being positive by direct MALDI-TOF/MS, but the identification protocol failed, although all the cases were identified using routine MALDI-TOF/MS. It is important to mention that of those cases, almost 50% were polymicrobial infections. From those eight samples, routine MALDI-TOF/MS managed to successfully identify all the cases; however, direct MALDI-TOF/MS failed to identify any of them. Those results can be explained by the aberrant protein spectrum produced when MALDI-TOF/MS is used with polymicrobial samples, resulting in a mixture of several profiles, yielding MALDI-TOF/MS unable to identify the bacteria from its database [38,39].

So, as seen in the previous study described, an important drawback of this method is still the lack of positive results when using MALDI-TOF/MS to identify polymicrobial infections. For those cases of fungal infections, routine MALDI-TOF/MS remains a better diagnostic tool than direct MALDI-TOF/MS.

Same in other studies, the most emphasized aspect when using direct MALDI-TOF/MS as a tool to identify the pathogens in PJI was the shortening of time required to obtain positive results. The earlier we can identify the pathogen, the more precisely we can initiate targeted antibiotic treatment to reduce the development of resistant strains. Results from the presented study show that direct MALDI-TOF/MS pathogen identification from synovial fluid inoculated BCB reduced the time required to get organism identification to a median duration of 12.7 hours. This duration was significantly lower than the median duration of 39.5 hours for routine MALDI-TOF/MS and 44.4 hours for conventional culture [39].

Species-level bacteria identification using MALDI-TOF/MS in PJI

Another study published by Peel et al. [40] in 2014 wanted to prove the use of MALDI-TOF/MS in understanding PJI pathogenesis and diagnosis. The search for an accurate test to lower the false positive, and false negative rates is challenging because unfortunately, the most common pathogens are commensal skin organisms that are also frequent specimen contaminants like in the case of Coagulase-negative Staphylococcus species [41,42]. A total of 260 arthroplasty cases were studied, from which 178 PJIs and 82 aseptic failures, yielding 770 organisms to be analyzed. MALDI-TOF/MS managed to identify 455 organisms (59%) in 197 cases (123 PJI and 74 aseptic failures), with 89% of them identified at the species level. Knowing that not all laboratories are routinely identifying organisms such as coagulase-negative staphylococci at the species level, the study aimed to verify if using MALDI-TOF/MS for species detection alongside other rapid identification tests will yield better results in the overall identification of true pathogens and contamination specimens. The high number of microorganisms detected from the aseptic failure samples could also indicate a high misdiagnose rate.

The study shows good results for the use of MALDI-TOF/MS as an auxiliary test for rapid bacterial identification in PJI cases. They showed that the profile of the isolates differs when they are true pathogens or contaminants. Knowing that some organisms like Staphylococcus aureus or Staphylococcus caprae were always associated with PJI, Staphylococcus epidermidis being equally distributed between pathogens and contaminants (17% and 19%) and most streptococcal specimens were pathogens (12% vs. 4% contaminants), MALDI-TOF/MS testing can potentially guide clinical decision in the case of single positive culture results when the differential diagnosis is difficult [40]. The downside of the study is that MALDI-TOF/MS could not reliably identify some species and that the small numbers of some identified species could influence the pathogen/contaminant ratio. Considering that two positive cultures with the same microorganism are necessary for PJI diagnosis using Musculoskeletal Infection Society (MSIS) or Infectious Diseases Society of America (ISDA) criteria, MALDI-TOF/MS could be used and the results could be compared to other microbiological tests.

Alpha-defensin detection using MALDI-TOF/MS in PJI

We want to draw attention to an interesting study led by Iorio et al. [43] in 2021 who presented a different perspective on the use of MALDI-TOF/MS. Instead of using this method to try to identify the pathogens like the other three studies described above, they focused on assessing the accuracy of this technique for alpha defensin detection in synovial fluid of patients who needed to undergo revision surgery for arthroplasty failure. The study hypothesized that alpha defensin detection through MALDI-TOF/MS examination could be a highly sensitive and specific test in diagnosing PJI when using MSIS criteria. The use of alpha defensin as a synovial marker of PJI is not new to the literature; currently, two types of detection systems are commercially available, but with high costs and maybe some uncertain accuracy.

Data from this study were compared with those already available detection methods like the ELISA laboratory-based test and lateral flow test (Synovasure), emphasizing the pros and cons of each type. Both of the tests are expensive, with superior overall diagnostic accuracy of the ELISA, but requiring a dedicated laboratory for it and the long time to get results are still the most important concerns [20].

The study included 138 cases of arthroplasty revision: 59 cases of PJIs and 79 cases of aseptic failures, with the two groups classified according to the 2018 MSIS criteria. Using MALDI-TOF/MS to detect alpha defensin in the synovial fluid, they managed to correctly identify 55 of 59 cases with PJIs and 76 of 79 patients with aseptic failure of arthroplasty. The data concluded with the MALDI-TOF/MS examination overall accuracy being 94.9%. With sensitivity as high as 93% and a specificity of 96%, the test showed promising results in identifying PJIs [43].

The specificity and sensitivity of already available Synovasure and ELISA assays were described in the study by bringing into discussion several recent studies. Although initial studies found high accuracy for both tests, more recent results show a significantly lower sensitivity of only 67% and specificity of 94% in the case of the lateral flow test [44].

The use of MALDI-TOF/MS assay for alpha defensin detection in synovial fluid turned out to be a promising diagnostic tool for PJIs with high sensitivity and specificity. The test is cheap, the technology to sustain this kind of assay is already widely available in the laboratories of hospitals and another major advantage is represented by the very short period (20 minutes) to get a positive result, meaning that the treatment plan can be adjusted in the operating room.

For an easier understanding, sensitivities and specificities of MALDI-TOF/MS test when used for PJI detection or bacterial identification were presented in Table 1.

**Table 1:** Sensitivity and specificity of MALDI-TOF/MS from all studies included in the review. *Three patients were diagnosed with periprosthetic infection involving two different joints. PJI, periprosthetic joint infection; AL, aseptic loosening; MALDI-TOF/MS, matrix-assisted laser desorption ionization time-of-flight mass spectrometry

Study	Population	Sample used	Targeted proteins for bacterial identification or PJI detection	Sensitivity	Specificity
Beguiristain et al. (2023) [38]	107 patients (36 PJI and 71 AL)	Sonication fluid inoculated in blood culture bottles	Bacterial proteins	69%	94%
Kuo et al. (2020) [39]	77 patients (77 PJI and 80 cases*)	Synovial fluid inoculated in blood culture bottles	Bacterial proteins	80%	-
Peel et al. (2014) [40]	197 patients (123 PJI and 74 AL)	Positive tissue, synovial fluid, and sonication fluid cultures	Bacterial proteins	59%	-
Iorio et al. (2021) [43]	138 patients (59 PJI and 79 AL)	Synovial fluid	Human alpha defensin - antimicrobial peptide	93%	96%
Harris et al. (2010) [41]	79 patients (79 PJI and 158 bacterial isolates)	Tissue and synovial fluid cultures	Bacterial proteins	100%	-

The major limitation of this review article is the low number of cohort studies conducted regarding the utility of MALDI-TOF/MS in prosthetic joint infections. Another consequence of this limitation is reflected in the inconsistency of the partial results when presented in tabular form. Although some studies tried to use this method for PJI diagnosis, the accuracy of the test is still not high enough to be routinely used as a singular test, the current state of the technology being more suitable as an ancillary test for the usual diagnostic methods to improve their speed and accuracy. Our study evaluated different perspectives regarding the importance of MALDI-TOF/MS in the infectious pathology of orthopedic implants to highlight its strengths and limitations to encourage further research in this direction. To summarize our review of this method and its current state when used as a diagnostic tool in PJI we further exposed its main advantages and disadvantages in Table 2.

**Table 2:** Main advantages and disadvantages of MALDI-TOF/MS in PJI. PJI, periprosthetic joint infection; MALDI-TOF/MS, matrix-assisted laser desorption ionization time-of-flight mass spectrometry

Pros	Cons
More than three times faster than other techniques when using direct MALDI-TOF/MS	Direct technique has lower sensitivity than routine MALDI-TOF/MS
Very cheap (0.50$ per specimen) [37]	Best accuracy is obtained when used on pure bacterial isolates (this method requires another step and negates the time efficiency advantage)
Technology is already widely available	Low sensitivity for polymicrobial or fungal infections when using direct MALDI-TOF/MS (in this case, routine technique is more reliable)
Can also detect other PJI biomarkers, not only bacterial proteins	Differential centrifugation of the samples required to increase accuracy of direct MALDI-TOF/MS
Can differentiate bacterial pathogens from contaminants	At the moment, the accuracy of this method is not high enough to be used as a sole diagnostic tool
Can identify bacteria to the species level	Only a few studies have been published about the utility of this method in orthopedic implants infectious pathology - more research is needed
Used as an auxiliary test can improve bacterial identification and PJI detection accuracy	
Room for improvement with the growing spectral database	
User-friendly procedure	

## Conclusions

PJI requires fast identification of causative bacteria. This step is very important in the evolution and prognosis of the patient. By highlighting the results from the most cited and recent studies we showed that MALDI-TOF/MS technology has an invaluable role in rapid bacterial identification and diagnosis of PJI. With the evolving spectra database and methods of implementing MALDI-TOF/MS in PJI diagnosis, we see great potential for this technology in the future. The great specificity and sensitivity combined with MALDI-TOF’s rapid use and reduced costs make it an appealing option for clinical laboratories, that could support orthopedic surgeons in improving the diagnostic accuracy and overall treatment success rate of arthroplasty revision pathology.
